# Selected Papers from the pHealth 2021 Conference, Genoa, Italy, 8–10 November 2021

**DOI:** 10.3390/jpm13081213

**Published:** 2023-07-31

**Authors:** Bernd Blobel, Mauro Giacomini

**Affiliations:** 1 Medical Faculty, University of Regensburg, 93053 Regensburg, Germany; 2 Faculty European Campus Rottal-Inn, Deggendorf Institute of Technology, 94469 Deggendorf, Germany; 3 First Medical Faculty, Charles University of Prague, Stare Mesto 110 00, Czech Republic; 4 DIBRIS, University of Genoa, 16145 Genoa, Italy; mauro.giacomini@dibris.unige.it

This Special Issue of the *Journal of Personalized Medicine* presents extended versions of selected contributions to pHealth 2021, the 18th International Conference on Wearable Micro and Nano Technologies for Personalized Health, held on 8–10 November 2021 in Genoa, Italy. The original papers have been published in the IOS Press Studies in Health Technology and Informatics 2021, volume 285 (https://ebooks.iospress.nl/volume/phealth-2021-proceedings-of-the-18th-international-conference-on-wearable-micro-and-nano-technologies-for-personalized-health-810-november-2021-genoa-italy) (URL accessed on 15 June 2023).

The 2021 edition of pHealth emphasized the interrelated aspects of advanced pHealth, i.e., personalized, participative, preventive, predictive, precision medicine (5P medicine) in health and social services. In that context, mobile technologies, micro–nano–bio smart systems, artificial intelligence and robotics, data management and analytics, machine learning and deep learning for personalized health, the Health Internet of Things (HIoT), systems medicine, public health, and virtual care are of interest. Those new technologies create new potential risks for security, privacy, and safety, resulting in new challenges for meeting ethical and trustworthiness requirements of systems, partners, and processes. Bernd Blobel as the long-term Chair of the pHealth conferences’ Scientific Program Committee as well as of the pHealth Steering Committee has checked and edited every paper invited for publication in the MDPI *JPM* pHealth 2021 Special Issue before giving the green flag for formal submission. Mauro Giacomini, as Chair of the pHealth 2021 Local Organizing Committee, has managed the review process, performed by at least two independent international experts.

The book starts with an introduction into the ongoing transformation of health and social care including the related organizational, methodological, and technological paradigm changes. For designing and managing ethical and intelligent transformed health ecosystems, the comprehensive and correct formal representation complex, dynamic, interdisciplinary ecosystems with their knowledge spaces is inevitable. Regarding ethical, legal, security, and privacy aspects, the system’s policy domain and their aforementioned subdomains must be especially addressed. Therefore, the paper specifically discusses the deployment of ontologies for representing ecosystems and their domains, hereby also considering newly standardized ontologies for representation and management of ethically driven robotics and automation systems. Thereafter, knowledge representation and management for semantic data integration is discussed in the context of practical solutions for biobanks. As the new technologies and methodologies are not just necessary for developing and running transformed health and social care ecosystems, but are also inevitable for properly including the current and potential actors, the introductory chapter of the book concludes with a paper on didactic concepts for digital learning in care settings.

The second chapter presents two papers tackling the deployment of mobile technologies for pHealth. The first discusses and compares different approaches to learning systems for managing ambulant stress detection. The second one studies the deployment of mobile applications for improving care processes, especially the communication between care providers in pediatrics.

The third chapter discusses the deployment of machine learning, artificial intelligence, and automation in transformed health and social care ecosystems with concrete solutions. The first paper focuses on Natural Language Processing, and the last one on unsupervised learning for automatically analyzing notes and electronic medical records. The second paper analyzes processes optimization with machine learning. The papers that follow investigate the deployment of Chatbots in the context of behavioral health, the deployment of machine learning to analyze the side effects of CVD interventions, the intelligent analysis of COVID-19 pneumonia cases using Hybrid Bayesian Networks, and, finally, the use of decision support systems.

The last chapter addresses security, privacy, safety, and trust issues changing their characteristics as well as occurrence and importance in the context of the transformation of health and social care ecosystems. First, the implementation of standard-based security and privacy in genomic information systems is discussed at length. Thereafter, a new methodology is presented for assessing privacy and trust in eHealth. The penultimate paper of this volume addresses the importance of data democratization for the advancement of data sharing at a national and European level, as well as globally, while the last paper offers a risk prediction methodology in cardiac surgery.

The editors thank all authors and reviewers for their important contribution to the success of this volume. Furthermore, they are deeply indebted to the MDPI *Journal of Personalized Medicine* and its Editorial Office, and especially to Penny Su, for the valuable continuous support. Without all those efforts, this volume would not have been possible.

